# Gene expression associated with unfavorable vaginal bleeding in women using the etonogestrel subdermal contraceptive implant: a prospective study

**DOI:** 10.1038/s41598-024-61751-7

**Published:** 2024-05-14

**Authors:** Flávia R. Torelli, Raquel M. Rodrigues-Peres, Ilza Monteiro, Iscia Lopes-Cendes, Luis Bahamondes, Cássia R. T. Juliato

**Affiliations:** 1 Department of Obstetrics and Gynecology, School of Medical Sciences, University of Campinas (UNICAMP), Campinas, SP Brazil; 2 Department of Translational Medicine, School of Medical Sciences, University of Campinas (UNICAMP), Campinas, SP Brazil; 3 Departamento de Tocoginecologia, Faculdade de Ciências Médicas, Universidade Estadual de Campinas – UNICAMP, Rua Alexander Fleming 101, Campinas, SP 13083-881 Brazil

**Keywords:** Etonogestrel implant, Contraception, Uterine bleeding, Genes, Implanon®, Gene expression, Medical genetics, Reproductive signs and symptoms

## Abstract

To evaluate gene expression associated with unfavorable vaginal bleeding in users of the Etonogestrel (ENG) contraceptive implant. Prospective study involving 100 women who intended to use the ENG implant. Exclusion criteria included abnormal uterine bleeding, inability to attend a 1-year follow-up, and implant removal for reasons unrelated to vaginal bleeding or loss of follow-up. We obtained endometrial biopsies before implant placement and assessed the expression of 20 selected genes. Users maintained a uterine bleeding diary for 12 months post-implant placement. For statistical analysis, we categorized women into those with or without favorable vaginal bleeding at 3 and 12 months. Women with lower CXCL1 expression had a 6.8-fold increased risk of unfavorable vaginal bleeding at 3 months (OR 6.8, 95% CI 2.21–20.79, p < 0.001), while those with higher BCL6 and BMP6 expression had 6- and 5.1-fold increased risks, respectively. By the 12-month follow-up, women with lower CXCL1 expression had a 5.37-fold increased risk of unfavorable vaginal bleeding (OR 5.37, 95% CI 1.63–17.73, p = 0.006). Women with CXCL1 expression < 0.0675, BCL6 > 0.65, and BMP6 > 3.4 had a higher likelihood of experiencing unfavorable vaginal bleeding at 3 months, and CXCL1 < 0.158 at 12 months. Users of ENG contraceptive implants with elevated BCL6 and BMP6 expression exhibited a higher risk of breakthrough bleeding at the 3-month follow-up. Conversely, reduced CXCL1 expression was associated with an elevated risk of bleeding at both the 3 and 12-month follow-ups.

## Introduction

The Etonogestrel (ENG) subdermal contraceptive implant is a widely used long-acting reversible contraceptive (LARC) method^1^. Despite its high contraceptive efficacy and impressive continuation rate, exceeding 80% after the first year of use^2^, the primary reason for discontinuation is the occurrence of an adverse uterine bleeding pattern. This pattern is characterized by either prolonged bleeding or spotting episodes lasting more than 14 days within a 90-day reference period, or frequent bleeding involving more than five bleeding or spotting episodes within the same reference period^3^.

Unlike menstrual bleeding in eumenorrheic women, which results from a decline in serum progesterone levels, the bleeding associated with progestogen-only contraceptive implants occurs sporadically and irregularly. This irregularity is linked to a distribution of delicate and superficial vessels within the endometrium^4,5^.

Initial studies exploring the effects of subcutaneously administered progestogen-releasing implants on the endometrium revealed endometrial atrophy through ultrasound observations^6^. Subsequent research reported the absence of endometrial tissue in the majority of endometrial biopsies^7^. However, it's crucial to note that atrophy isn’t the primary cause of the irregular bleeding associated with progestogen use. Women experiencing uterine bleeding while using contraceptive implants with progestogens display fragility, alongside dilation and congestion of vessels within the subepithelial endometrium^8,9^.

Several factors contribute to this bleeding phenomenon, including increased expression of matrix metalloproteinase (MMP) 1, which initiates the breakdown of the extracellular matrix (ECM), leading to the detachment of endometrial tissue^10–12^.

Another proposed mechanism involves heightened expression of angiopoietins, which inhibit factors stabilizing blood vessels, thereby increasing vascular permeability and branching^13^. The upsurge in free radicals resulting from progestogen use may also contribute to increased vessel bleeding. Moreover, the presence of thrombin due to irregular bleeding can establish a vicious cycle, promoting further thrombin production, weakening vasculature, and perpetuating bleeding^14^. Thrombin also binds to PAR-1 receptors expressed by endometrial stromal cells, encouraging abnormal angiogenesis and inflammation, ultimately elevating the levels of vascular endothelial growth factor (VEGF) and interleukin-8 (IL-8)^15^. Furthermore, thrombin triggers the release of MMP-1 from endometrial stromal cells, selectively degrading interstitial collagens, and MMP-3, which subsequently breaks down various other ECM proteins while activating secreted MMP zymogens^15^.

Thus, an increase in angiogenesis, MMP activity, and immune and inflammatory factors are interconnected with endometrial instability. Several genes play a pivotal role in orchestrating this intricate process. A study involving human endometrial stromal cells revealed the modulation of 65 genes by synthetic and natural progesterone in a microarray analysis. Among these, 62% and 44% of genes were altered by the utilization of medroxyprogesterone acetate (MPA) or ENG, respectively. These genes significantly contribute to endometrial inflammation, angiogenesis, and bleeding^16^.

The primary objective of our study is to establish the correlation between the presence of favorable vaginal bleeding at 3- and 12-months following ENG contraceptive implant placement and the expression of selected genes. Our secondary objective is to determine an expression value threshold for genes associated with unfavorable vaginal bleeding.

## Materials and methods

### Study design and location

We conducted a prospective cohort study at the Department of Obstetrics and Gynaecology, Faculty of Medical Sciences, University of Campinas, Campinas, SP, Brazil, from May 2021 to September 2022. The Ethics Committee of the University of Campinas (UNICAMP) approved the study protocol (CAAE: 37320720.3.0000.5404) and all participants signed an informed consent form before entering in the study. We confirm that all research was performed in accordance with relevant guidelines/regulations and in accordance with the Declaration of Helsinki.

### Participants

We included a convenience sample of 100 women aged between 18 and 45 years who expressed a desire to use the ENG subdermal implant (Implanon NXT^®^; Organon, Oss, The Netherlands) for contraception and displayed no contraindications to the method^17^. Women with a history of abnormal uterine bleeding (AUB)^18^, including endometriosis, endometrial polyps, uterine fibroids (leiomyomas), and adenomyosis were excluded, women in use of hormonal method of contraception, as well as those unable to attend a 1-year follow-up. Participants who had the implant removed for reasons unrelated to unfavorable vaginal bleeding within one year of placement were also discontinued from the study.

### Procedures

All participants underwent a comprehensive gynecological examination immediately before ENG implant insertion, during which an endometrial biopsy was obtained using exclusively a Pipelle of Cornier^®^ in the first menstrual phase (until 7 days of the cycle). These specimens were then stored at − 80 °C until analysis. Following the gynecological examination, the ENG implant was placed. Participants were instructed to maintain a vaginal bleeding diary throughout 12 months after device placement. Instances of vaginal bleeding were characterized as any bloody vaginal discharge necessitating the use of pads or tampons, while spotting was described as any bloody vaginal discharge that did not require protective measures^19^. Regular telephone contacts occurred every three months, with a comprehensive face-to-face assessment at the 12-month mark to collect and assess all menstrual diaries.

### Gene selection

The samples were stored at − 80 °C and subjected to a gene selection process targeting genes related to menstrual bleeding. These genes encompassed functional pathways such as immune and inflammatory response, angiogenesis, regulation of apoptotic processes, and matrix metalloproteinases (MMPs)^19–28^. Using the DAVID Bioinformatics Database (https://david.ncifcrf.gov), the most relevant 20 genes were selected and categorized into four distinct groups: immune response, inflammatory response, angiogenesis, and MMPs (Supplementary Information).

### RNA extraction

For the targeted genes, RT-qPCR employed pre-designed and validated hydrolysis probes obtained from Thermo Fisher Scientific™. Serial dilutions were used to create a standard curve to determine the optimal RNA quantity for each qPCR reaction, which was determined to be 25 ng. Three reference genes were utilized: ACTB, GAPDH, and PRDM4. The first two genes, widely recommended by the manufacturer as qPCR controls, were supplemented by PRDM4, chosen based on its stability in endometrial samples after comprehensive testing^29^.

The reverse transcription process employed the SuperScript™ IV VILO™ Master Mix with ezDNase™ Enzyme (Cat# 11766050, Thermo Fisher Scientific). Each sample consisted of 2.5 µg of the RNA reaction mix, strictly following the manufacturer’s guidelines. This process included DNAse enzyme treatment at 37 °C for 2 min to ensure the absence of DNA contamination in reverse transcription. The cDNA was generated through steps including primer annealing, reverse transcription, and enzyme inactivation. The resulting cDNA was promptly utilized in qPCR reactions or stored at − 80 °C.

For qPCR, the TaqMan™ Fast Advanced Master Mix (Cat# 4444965, Thermo Fisher Scientific) was employed, with each 20-µl reaction containing 25 ng cDNA. Following the manufacturer's hydrolysis probe protocol, samples were run in duplicate on 96-well plates using the 7500 Real-Time PCR System (Cat# 4377354, Thermo Fisher Scientific). The ensuing analysis utilized the Relative Quantification application available on the Thermo Fisher ConnectTM website (version 1.1). Replicates with more than a 0.5 cycle variation in Cqs were excluded, and interplate variation was mitigated by employing a consistent known sample for calibration.

### Statistical analysis

Participants were categorized into groups experiencing either favorable or unfavorable vaginal bleeding at 3 and 12 months. The concept of favorable bleeding encompassed reduced menstrual flow (amenorrhea, infrequent bleeding), while unfavorable bleeding was characterized by increased or unpredictable flow (defined as frequent, prolonged, and irregular bleeding) as delineated by Hankel and Belsey. To explore factors linked to unfavorable bleeding at 3 and 12 months, both simple and multiple logistic regression analyses were conducted, with variable selection employing a stepwise criterion. Statistical significance was set at 5%. Mann–Whitney analysis was utilized to associate genes with bleeding patterns at 3 and 12 months. In instances where genes demonstrated a trend (significant level up to 1%) or statistically significant difference, ROC curves were applied to determine predictive gene expression thresholds.

We calculated the sample power considering proportion values in each group setting the significance level alpha at 5% (Type I error), and the size of groups in the current sample (n = 59 favorable and n = 37 unfavorable in 3 months of follow up and n = 50 favorable and n = 40 unfavorable in 12 months follow up). Calculation of the sample size considering an 80% power and a 5% significance level.

## Results

**Figure 1:**
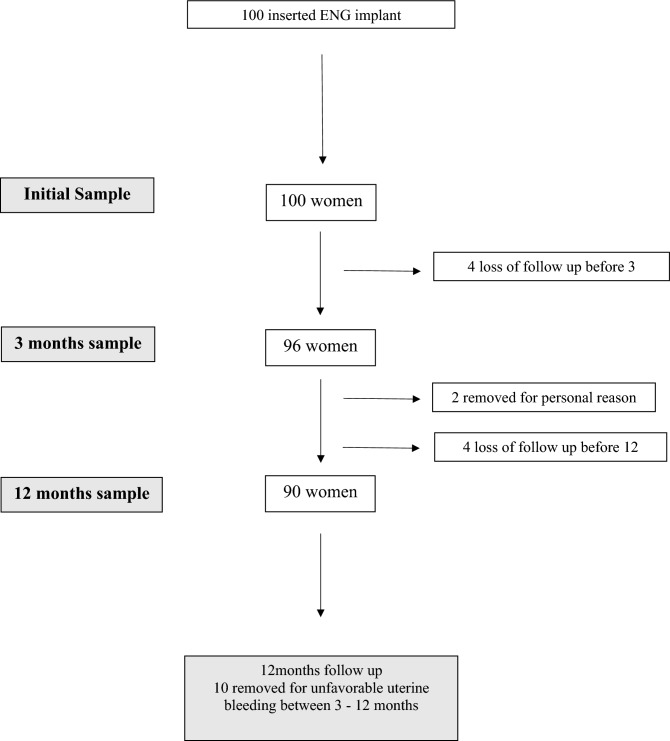
Flow chart of the women included in the study.

Figure 1 presents details regarding participant selection. A total of 100 ENG implants were inserted. During the follow-up period, contact was lost with 4 participants, resulting in a total sample of 96 women available at the 3rd month of analysis. Between the 3rd and 12th months, an additional 4 participants were lost to follow-up: 2 removed the device for personal reasons and were consequently excluded, while the other 2 did not return for their appointments. This led to a final count of 92 women available for the 12-month follow-up analysis. Within the 3- to 12-month period, a total of 10 women chose to remove the device due to unfavorable bleeding.

At the 3-month follow-up, uterine bleeding profiles were categorized as favorable in 59 women (61.5%) and unfavorable in 37 women (38.5%). The mean age of the participants was 27.8 (± 6.4) years, with no significant statistical distinctions observed between the groups experiencing favorable and unfavorable bleeding (Table 1). There were no disparities noted in terms of ethnicity, years of schooling, BMI (kg/m^2^), parity, age at menarche, menstrual period duration, or prior cycle length before the study initiation.

**Table 1:** Characteristics of the women according to menstrual bleeding and ENG subdermal insertion time.

	Total (n = 100)	Follow up					
		3 meses (n = 96^#^)		P	12 meses (n = 90^##^)		P
		Favorable (n = 59)	No favorable (n = 37)		Favorable (n = 50)	No favorable (n = 40)	
Age (years) mean ± SD	27.8 ± 6.4	27.9 ± 6.9	27.6 ± 5.8	0.266*	27.8 ± 6.8	28.5 ± 6.3	0.256*
< 20 years n(%)	8 (8%)	5 (8.5%)	3 (8.1%)		6 (12%)	1 (2.5%)	
20–29 -years n(%)	51 (51%)	33 (55.9%)	18 (48.6%)		22 (44%)	24 (60%)	
30–39 years n(%)	32 (32%)	17 (28.1%)	16 (43.2%)		20 (40%)	13 (32.5%)	
≧ 40 years n(%)	4 (6%)	4 (6.8%)	0		2 (4%)	2 (5%)	
Ethnicity (n = 100)							
White	45 (45%)	25 (42.4%)	17 (45.9%)	0.731**	20 (40%)	21 (52.5%)	
Not white	55 (55%)	34 (57.6%)	20 (54%)		30 (60%)	19 (47.5%)	0.237**
Schooling (n = 100) years (mean ± SD)	12.3 ± 2.6	12.2 ± 2.5	12.3 ± 2.7	0.893***	12.3 ± 2.6	12 ± 2.5	0.408***
BMI (kg/m^2^) (n = 100) mean ± SD	28.9 ± 6.4	29.3 ± 6.2	28.1 ± 6.7	0.387***	29.3 ± 6.8	28.5 ± 6.2	0.62***
Parity mean ± SD (n = 100)	1.1 ± 1.3	1.1 ± 1.3	1.2 ± 1.3	0.52***	1.0 ± 1.1	1.4 ± 1.5	0.241***
Age at menarche years mean ± SD (n = 97)	12.3 ± 1.6	12.4 ± 1.7	12.1 ± 1.6	0.471***	12.4 ± 1.8	12.1 ± 1.5	0.352***
Hysterometry centimeters (n = 58)	7.6 ± 0.5	7.6 ± 0.5	7.6 ± 0.6	0.884***	7.7 ± 0.6	7.5 ± 0.4	0.471***
Duration of menstrual period (days) (n = 99)	4.7 ± 1.3	4.5 ± 1.2	4.8 ± 1.4	0.256***	4.6 ± 1.2	4.7 ± 1.5	0.925***
Cycle length (days) (n = 99)	30.9 ± 9.6	31.1 ± 9.1	30.5 ± 10.9	0.524***	30.7 ± 9.8	29.6 ± 4.3	0.617***

*BMI* body mass index.

*Exato de Fischer; **X^2^; ***Mann–Whitney test; ^#^4 loss of follow up before 3 months; ^##^2 removed for personal reason and 4 loss of follow up before 12 months.

After the results, the sample power was calculated, with the genes CXCL1 < 0.0675 having a power of 0.695, BMP6 > 3.4 with a power of 0.760, and BCL2 > 0.65 with a power of 0.746 to distinguish between women with favorable and unfavorable bleeding at the 3-month follow-up. For the gene CXCL2, the power was 0.963 to differentiate women with favorable and unfavorable bleeding at the 12-month follow-up.

### Gene expression analysis

Regarding gene expression, BCL6 exhibited higher expression (p = 0.035), whereas CXCL1 showed lower expression among women experiencing unfavorable bleeding during the third-month follow-up (p = 0.05). Additionally, BMP6 displayed a trend toward elevated expression among women with unfavorable bleeding (p = 0.063) (Table 2). Multiple regression analyses revealed that women with decreased CXCL1 expression had a 6.8-fold higher likelihood of experiencing unfavorable vaginal bleeding within 3 months (OR 6.8, 95% CI 2.21–20.79, p < 0.001). Similarly, women exhibiting higher expression of BCL6 and BMP6 faced sixfold and 5.1-fold increased odds of unfavorable vaginal bleeding, respectively (Table 3).

**Table 2:** Gene expression according to favorable bleeding and follow up of ENG implant insertion.

Genes expression	Total	Follow up					
		3 months			12 months		
		Favorable bleeding (n = 59)	Unfavorable bleeding	P	Favorable bleeding (n = 50)	Unfavorable bleeding	P
	Mean ± SD	Median (Q1-Q3)			Median (Q1-Q3)		
*BCL6* (n = 100)	2.01 ± 1.96	1.0(0.6–1.8)	1.4 (0.8–3.9)	0.035	0.9 (0.6–2.3)	1.2 (0.7–3.8)	0.383
*BMP6* (n = 99)	5.27 ± 3.95	3.8 (2.5–6.1)	4.9 (3.6–9.1)	0.063	4.6 (2.8–7.3)	4.2 (2.3–6.1)	0.618
*C3* (n = 100)	0.99 ± 1.1	0.5 (0.2–1.3)	0.7 (0.4–1.5)	0.249	0.5 (0.2–1.5)	0.7 (0.3–1.4)	0.997
*CCL2* (n = 100)	1.54 ± 1.32	1.2 (0.7–2)	1.1 (0.7–1.5)	0.593	1.3 (0.7–1.8)	1 (0.6–1.6)	0.289
*CCL3* (n = 99)	3.53 ± 4.29	1.5 (1.0–4.9)	2.2 (1.1–4.4)	0.547	2.1 (1.1–5)	1.6 (0.9–4.9)	0.591
*CCL4* (n = 100)	2.81 ± 3.04	1.6 (1.0–3.0)	2 (1.1–3.8)	0.486	1.9 (1–3)	1.9 (1–3.5)	0.783
*CCR1* (n = 99)	3.46 ± 3.27	2.1 (1.2–3.6)	2 (1.5–5.1)	0.412	2.3 (1.6–3.6)	1.9 (1.2–4.6)	0.433
*CD40* (n = 100)	1.51 ± 1.09	1.2 (0.8–2.2)	1.1 (0.9–1.5)	0.52	1.5 (0.9–2.3)	1.1 (0.7–1.5)	0.077
*CXCL1* (n = 100)	0.33 ± 0.92	0.1 (0.05–0.2)	0.1 (0–0.1)	0.050	0.1 (0–0.2)	0.1 (0–0.1)	0.079
*CXCL10* (n = 100)	1.66 ± 1.88	1.0 (0.7–2.0)	1.0 (0.7–1.6)	0.83	0.9 (0.6–1.8)	1.2 (0.7–2)	0.326
*CXCL12* (n = 100)	10.62 ± 7.8	8.9 (5.0–8.9)	6.9 (4.7–14)	0.59	9 (4.8–14.2)	8.7 (5.1–14.9)	0.994
*CXCL9* (n = 99)	1.98 ± 4.85	0.8(0.4–1.4)	0.7 (0.4–1.3)	0.771	0.7 (0.4–1.7)	0.8 (0.4–1.4)	0.984
*IL15* (n = 99)	4.3 ± 5.3	1.6(1.1–4.2)	2 (1.1–6)	0.332	1.9 (1.3–6)	1.7 (1.1–5.1)	0.779
*IL17A* (n = 98)	0.21 ± 1.1	0.05 (0.0–0.1)	0 (0–0.1)	0.187	0 (0–0.1)	0.1 (0–1)	0.585
*MMP19* (n = 99)	16.34 ± 16.14	11.5 (8.5–20)	11 (8–15.5)	0.675	13 (8.9–21.1)	10.6 (8.1–15.7)	0.231
*MMP2* (n = 100)	5.23 ± 5.16	3.5 (2.4–5.4)	3.6 (2.6–5.3)	0.69	3.8 (2.6–6.3)	3.6 (2.5–5.1)	0.573
*SYK* (n = 99)	0.74 ± 0.39	0.6 (0.5–0.9)	0.6 (0.5–0.9)	0.921	0.6 (0.4–1)	0.7 (0.5–1)	0.869
*TIMP1* (n = 93)	0.43 ± 0.40	0.3 (0.2–0.5)	0.3 (0.2–0.5)	0.634	0.5 (0.3–2.9)	0.3 (0.2–0.5)	0.372
*TIMP2* (n = 98)	5.22 ± 3.92	3.8 (2.8–6.0)	3.8 (2.8–6)	0.484	4.4 (3.4–6.6)	3.8 (3–5.6)	0.249
*TNFRSF11B* (n = 99)	10.51 ± 29.63	2 (1.1–3.9)	2 (1.1–3.9)	0.457	1.9 (1.1–5.3)	1.8 (0.9–3.72)	0.473
Imune response (n = 100)	48.43 ± 40.46	39.5 (26–62.1)	39.5(26–62.3)	0.611	39.7 (24–68.1)	36.6 (25.3–61.2)	0.665
Inflammatory response (n = 100)	51.17 ± 40.96	41.8 (28.9–64.9)	41.8(28.9–64.9)	0.545	41.7 (25–72.6)	38.2 (27.3–64.9)	0.615
Angiogenesis (n = 100)	23.67 ± 21.24	16.3 (12.8–22.4)	16.3 (12.8–22.4)	0.795	19.3 (14.4–28)	16.6 (12.1–23.4)	0.217
Metalloproteinases (n = 100)	26.95 ± 24.60	19.3 (14.1–26.7)	19.3 (14.1–26.7)	0.772	21.8 (15.9–32.2)	19.9 (12.6–23.9)	0.188

Genes: BCL6 transcription repressor, *BCL6*; bone morphogenetic protein 6, *BMP6*; complement C3, *C3*; C–C motif chemokine ligand 2, *CCL2*; C–C motif chemokine ligand 3, *CCL3*; C–C motif chemokine ligand 4, *CCL4* ; C–C motif chemokine receptor 1, *CCR1*; CD40 molecule, *CD40*; C-X-C motif chemokine ligand 1, *CXCL1*; C-X-C motif chemokine ligand 9, *CXCL9*; C-X-C motif chemokine ligand 10, *CXCL10*; C-X-C motif chemokine ligand 12, *CXCL12*; interleukin 15, *IL15*; interleukin 17A, *IL17A*; matrix metallopeptidase 2, *MMP2*; matrix metallopeptidase 19, *MMP19*; spleen associated tyrosine kinase, *SYK*; TIMP metallopeptidase inhibitor 1, *TIMP1*; TIMP metallopeptidase inhibitor 2, *TIMP2*; TNF receptor superfamily member 11b, *TNFRSF11B*; actin beta, *ACTB*; glyceraldehyde-3-phosphate dehydrogenase, *GAPDH*; PR/SET domain 4, *PRDM4*.

**Table 3:** Gene expression and unfavorable vaginal bleeding regression analysis (3 months follow up).

Genes	Bivariate analysis			Multivariate analysis		
	O.R.*	CI 95% O.R.*	p-value	O.R.**	CI 95% O.R.**	P value
*BCL6*	1.202	0.968 – 1.491	0.095			
*BCL6* (ROC curve)	1.00 4.23	– 1.31–13.62	– 0.016	1.00 6.02	– 1.53–23.64	– 0.010
*BMP6*	1.099	0.989–1.222	0.079			
*BMP6* (ROC curve)	1.00 3.38	– 1.33–8.64	– 0.011	1.00 5.05	– 1.61–15.83	– 0.006
*C3*	1.192	0.827–1.718	0.347			
*CCL2*	0.771	0.524–1.135	0.188			
*CCL3*	0.987	0.896–1.087	0.790			
*CCL4*	1.012	0.887–1.156	0.856			
*CCR1*	1.044	0.922–1.181	0.499			
*CD40*	0.893	0.606–1.318	0.571			
*CXCL1*	0.820	0.482–1.395	0.464			
*CXCL1* (ROC curve)	1.00 2.86	– 1.22–6.69	– 0.015	1.00 6.78	– 2.21–20.79	– < 0.001
*CXCL10*	1.075	0.869–1.331	0.505			
*CXCL12*	0.989	0.937–1.045	0.703			
*CXCL9*	1.099	0.970–1.245	0.140			
*IL15*	1.020	0.944–1.101	0.619			
*IL17A*	1.434	0.533–3.857	0.475			
*MMP19*	0.980	0.949–1.012	0.215			
*MMP2*	0.992	0.916–1.075	0.851			
*SYK*	0.835	0.287–2.431	0.741			
*TIMP1*	0.630	0.192–2.063	0.445			
*TIMP2*	0.941	0.836–1.058	0.309			
*TNFRSF11B*	1.004	0.990–1.017	0.600			
Imune response	1.004	0.994–1.014	0.458			
Inflamatory response	1.004	0.994–1.014	0.241			
Angiogenesis	0.988	0.967–1.011	0.303			
Metalloproteinases	0.990	0.972–1.009	0.320			

*OR (*Odds Ratio*) = Risk ratio for unfavorable bleeding; (n = 37 and favorable bleeding n = 59). 95% CI OR = 95% confidence interval for hazard ratio.

**OR (*Odds Ratio*) = Risk ratio for unfavorable bleeding; (n = 37 and favorable bleeding n = 59). 95% CI OR = 95% confidence interval for hazard ratio. Stepwise variable selection criteria.

At the 12-month follow-up, endometrial expression of CXCL1 and CD40 showed a tendency toward lower levels among women with unfavorable bleeding (p = 0.079 and 0.077, respectively) (Table 2). Further regression analysis at 12 months revealed that women with reduced CXCL1 expression had a 5.37 times higher chance of experiencing unfavorable vaginal bleeding (OR 5.37, 95% CI 1.63–17.73, p = 0.006) (Table 4). Women who simultaneously exhibited CXCL1 expression lower than 0.0675, BCL6 expression lower than 0.65, and BMP6 expression lower than 3.4 had 15.43 times higher odds of experiencing breakthrough bleeding (OR 15.43, 95% CI 3.23–76.67, p < 0.001).

**Table 4:** Genes expression and vaginal bleeding regression analysis (12 months follow up).

Genes	Bivariate analysis			Multivariate analysis		
	O.R.*	CI 95% O.R.*	p-value	O.R.**	CI 95% O.R.**	P value
*BCL6*	1.051	0.850–1.299	0.648			
*BMP6*	0.969	0.871–1.079	0.567			
*C3*	0.893	0.612–1.303	0.558			
*CCL2*	0.927	0.675–1.271	0.637			
*CCL3*	0.977	0.886–1.076	0.633			
*CCL4*	0.973	0.849–1.114	0.690			
*CCR1*	1.021	0.902–1.156	0.740			
*CD40*	0.808	0.543–1.201	0.292			
*CD40* (ROC curve)	1.00 3.44	– 1.36–8.70	– 0.009			
*CXCL1*	0.906	0.574–1.430	0.671			
*CXCL1* (ROC curve)	1.00 7.07	– 2.19–22.87	– 0.001	1.00 5.37	– 1.63–17.73	– 0.006
*CXCL10*	1.103	0.884–1.377	0.384			
*CXCL12*	1.004	0.952–1.059	0.886			
*CXCL9*	1.042	0.951–1.142	0.374			
*IL15*	1.008	0.934–1.089	0.830			
*IL17A*	0.671	0.135–3.348	0.627			
*MMP19*	0.983	0.954–1.012	0.249			
*MMP2*	0.971	0.895–1.053	0.477			
*SYK*	0.996	0.328–3.026	0.994			
*TIMP1*	0.693	0.204–2.001	0.442			
*TIMP2*	0.944	0.842–1.058	0.325			
*TNFRSF11B*	1.001	0.848–1.077	0.911			
Imune response	1.001	0.991–1.011	0.915			
Inflamatory response	1.001	0.991–1.011	0.901			
Angiogenesis	0.987	0.966–1.009	0.243			
Metalloproteinases	0.989	0.970–1.007	0.233			

*OR (*Odds Ratio*) = Risk ratio for bleeding; (n = 58 no and n = 38 yes). 95% CI OR = 95% confidence interval for hazard ratio.

**OR (*Odds Ratio*) = Risk ratio for bleeding; (n = 47 no and n = 32 yes). 95% CI OR = 95% confidence interval for hazard ratio. Stepwise variable selection criteria.

### Receiver operating characteristic (ROC) analysis

**Figure 2:**
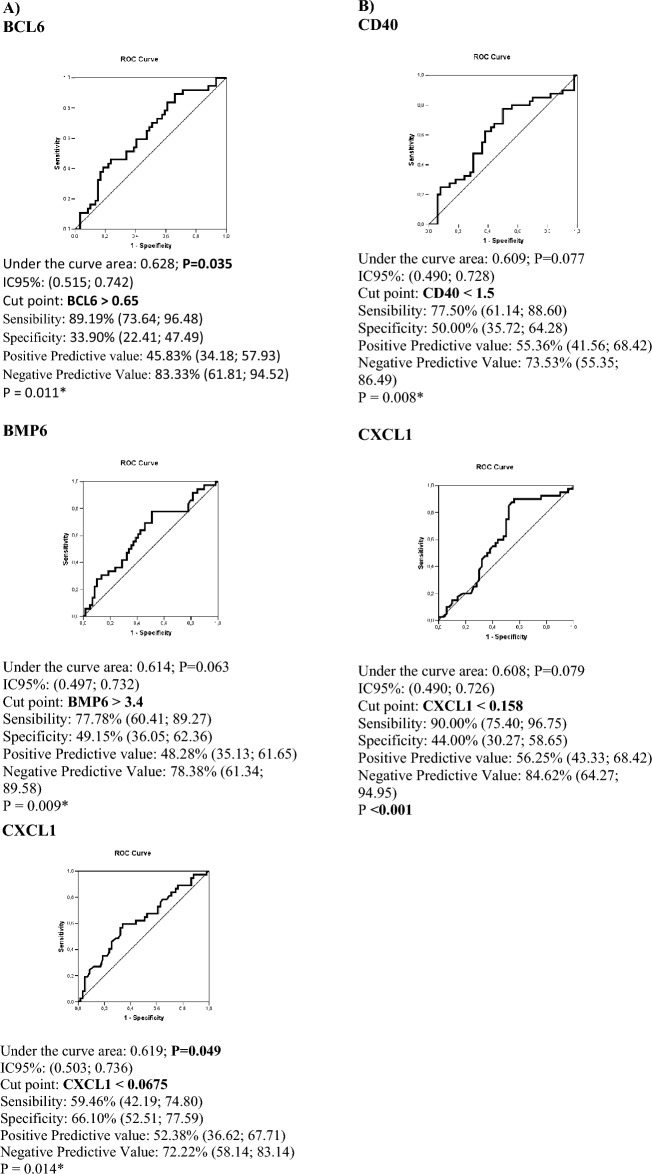
Results of receiver operating characteristic curve to evaluate a cut-off point for the values of significant genes as predictors of unfavorable bleeding at 3 and 12 months of follow up. (**A**) Genes (BCL6, BMP6 and CXCL1) associated with ETN implant users with unfavorable bleeding in 3 months of follow up. (**B**) Gene CD40 associated with ETN implant users with unfavorable bleeding in 12 months of follow up. *x^2^.

Figure 2 presents the results of Receiver Operating Characteristic (ROC) curve analysis aimed at establishing cutoff points for significant gene values as predictors of vaginal bleeding. A BCL6 value exceeding 0.65 demonstrated a substantial area under the curve, indicating a heightened likelihood of vaginal bleeding at 3 months. Conversely, CXCL1 values below 0.0675 were associated with an increased probability of vaginal bleeding at the 12-month mark (p = 0.049).

## Discussion

Our study uncovered a significant association between gene expression patterns and the occurrence of breakthrough bleeding in women using the ENG implant. Breakthrough bleeding, a common concern in contraceptive users, has been inadequately understood in terms of its underlying mechanisms. Our research aimed to shed light on this phenomenon by investigating the expression of specific genes related to endometrial stability and bleeding patterns^25^.

At the 3-month follow-up, we observed that ENG-implant users with elevated BCL6 gene expression had a substantially increased likelihood of experiencing breakthrough bleeding. The BCL6 gene, located on chromosome 3q27.3, plays a crucial role in B cell maturation and has a profound impact on the immune system^30^. Notably, its upregulation has been associated with conditions such as endometriosis, infertility, and pre-eclampsia, which involve vascular complications^31,32^. Therefore, heightened BCL6 expression may provide insights into the bleeding experienced by ENG-implant users.

In addition to BCL6, the levels of two other genes, CXCL1 (with expression < 0.0675, p < 0.001) and BMP6 (with expression > 3.4, p = 0.006), showed potential connections to unfavorable bleeding patterns at the 3-month follow-up. A similar trend was observed for CXCL1 expression (< 0.158, p = 0.006) at the 12-month follow-up. The BMP6 gene, situated on chromosome 6p24.3, encodes a growth factor that binds to TGF-β receptors. Research emphasizes its importance in endometriosis, infertility, and its influence on other genes like GDNF, impacting progesterone availability, a known factor in breakthrough bleeding^33,34^. BMP6 expression also correlates with dysmenorrhea in young women, reflecting responses to menstrual cycle-related inflammation processes^35^.

Furthermore, the CXCL1 gene exerts a profound influence on inflammation, immune responses, and tumor progression^36^. It is closely linked with vascular endothelial growth factors (VEGFs) and mitogen-activated protein kinases (MAPKs) in decidual angiogenesis and arteriogenesis processes^35,37^. Elevated VEGF levels, associated with microvascular density in the endometrium, contribute to bleeding in progestogen users^38^. A previous study demonstrated that downregulation of CXCL1 can lead to adenomyosis development due to its interaction with STAT3^39^. These findings underscore the significance of BMP6 and CXCL1 expressions in uterine tissue pathology, potentially contributing to unfavorable bleeding associated with the contraceptive method under evaluation.

Our study also revealed that the simultaneous involvement of CXCL1, BCL6, and BMP6 genes amplified the likelihood of experiencing breakthrough bleeding. Furthermore, our findings identified specific threshold values: a BCL6 value exceeding 0.65 at the 3-month follow-up and CXCL1 values below 0.0675 at 12 months correlated with breakthrough bleeding occurrences. These findings, to the best of our knowledge, lack direct comparisons in existing literature.

A notable strength of our study lies in addressing the dearth of literature exploring the connection between genes and breakthrough bleeding. Enhancing our understanding of the physiopathology behind this adverse effect linked to contraceptive methods containing progestogens holds promise for improved guidance for users and the development of targeted treatment strategies. However, a limitation of our study is the absence of endometrial samples after 12 months of use due to endometrial atrophy resulting from the method. While clinical signs of endometritis were not observed, confirmatory tests were not conducted. Future studies focusing on cellular processes and integrating different metabolic pathways, such as gene expression and tissue metabolomics, will be crucial for a deeper understanding of the physiopathology of breakthrough bleeding. Future studies with suppression of BCL6 with Gonadotropin-Releasing Hormone analog for 2 months prior to ENG implant insertion and reviewing the percentage of unfavorable bleeding to support your findings can also be interesting.

In conclusion, our study highlights the association between heightened BCL6 and BMP6 gene expressions and the increased likelihood of unfavorable vaginal bleeding at the 3-month follow-up in ENG-implant users. Reduced CXCL1 expression is also linked to bleeding occurrences, with significantly higher odds at both 3 and 12 months. These findings contribute to our understanding of the mechanisms behind breakthrough bleeding in ENG-implant users and have implications for further research and clinical management.

### Supplementary Information

Supplementary Information.

## Data Availability

The datasets generated and/or analysed during the current study are available in the *UNICAMP research repository* (REDU), https://redu.unicamp.br/dataset.xhtml?persistentId=doi:10.25824/redu/F2XCW4.
