# Supplementary material for: Gene expression associated with unfavorable vaginal bleeding in women using the etonogestrel subdermal contraceptive implant: a prospective study
Source: Sci Rep. 2024 May 14;14:11062. doi: 10.1038/s41598-024-61751-7 (PMC11093992; doi:10.1038/s41598-024-61751-7)
Supplement: Supplementary file 1 — Supplementary Information. [file 41598_2024_61751_MOESM1_ESM.docx]

Supplemental material: qPCR targets and oligonucleotides information

| **Gene symbol** | **Entrez Gene ID** | **Taqman® Gene Expression Assay ID** |
| --- | --- | --- |
| *BCL6* | <http://www.ncbi.nlm.nih.gov/gene?term=604> | Hs00153368_m1 |
| *BMP6* | <http://www.ncbi.nlm.nih.gov/gene?term=654> | Hs01099594_m1 |
| *C3* | <http://www.ncbi.nlm.nih.gov/gene?term=718> | Hs00163811_m1 |
| *CCL2* | <http://www.ncbi.nlm.nih.gov/gene?term=6347> | Hs00234140_m1 |
| *CCL3* | <http://www.ncbi.nlm.nih.gov/gene?term=6348> | Hs00234142_m1 |
| *CCL4* | <http://www.ncbi.nlm.nih.gov/gene?term=6351> | Hs99999148_m1 |
| *CCR1* | <http://www.ncbi.nlm.nih.gov/gene?term=1230> | Hs00174298_m1 |
| *CD40* | <http://www.ncbi.nlm.nih.gov/gene?term=958> | Hs00374176_m1 |
| *CXCL1* | <http://www.ncbi.nlm.nih.gov/gene?term=2919> | Hs00236937_m1 |
| *CXCL9* | <http://www.ncbi.nlm.nih.gov/gene?term=4283> | Hs00171065_m1 |
| *CXCL10* | <http://www.ncbi.nlm.nih.gov/gene?term=3627> | Hs00171042_m1 |
| *CXCL12* | <http://www.ncbi.nlm.nih.gov/gene?term=6387> | Hs00171022_m1 |
| *IL15* | <http://www.ncbi.nlm.nih.gov/gene?term=3600> | Hs01003716_m1 |
| *IL17A* | <http://www.ncbi.nlm.nih.gov/gene?term=3605> | Hs00174383_m1 |
| *MMP2* | <http://www.ncbi.nlm.nih.gov/gene?term=4313> | Hs01548727_m1 |
| *MMP19* | <http://www.ncbi.nlm.nih.gov/gene?term=4327> | Hs00418247_g1 |
| *SYK* | <http://www.ncbi.nlm.nih.gov/gene?term=6850> | Hs00895377_m1 |
| *TIMP1* | <http://www.ncbi.nlm.nih.gov/gene?term=7076> | Hs00171558_m1 |
| *TIMP2* | <http://www.ncbi.nlm.nih.gov/gene?term=7077> | Hs00234278_m1 |
| *TNFRSF11B* | <http://www.ncbi.nlm.nih.gov/gene?term=4982> | Hs00900358_m1 |
| *ACTB* (reference gene) | <http://www.ncbi.nlm.nih.gov/gene?term=60> | Hs01060665_g1 |
| *GAPDH* (reference gene) | <http://www.ncbi.nlm.nih.gov/gene?term=2597> | Hs03929097_g1 |
| *PRDM4* (reference gene) | <http://www.ncbi.nlm.nih.gov/gene?term=11108> | Hs00183764_m1 |
